# Long-Term Voluntary Physical Exercise Exerts Neuroprotective Effects and Motor Disturbance Alleviation in a Rat Model of Parkinson's Disease

**DOI:** 10.1155/2019/4829572

**Published:** 2019-12-05

**Authors:** Wan-Ling Tsai, Hsin-Yung Chen, Ying-Zu Huang, Yuan-Hao Chen, Chi-Wei Kuo, Kai-Yun Chen, Tsung-Hsun Hsieh

**Affiliations:** ^1^Ph.D. Program for Neural Regenerative Medicine, College of Medical Science and Technology, Taipei Medical University and National Health Research Institutes, Taipei 11031, Taiwan; ^2^Taipei Neuroscience Institute, Taipei Medical University, Taipei 11031, Taiwan; ^3^Department of Occupational Therapy and Institute of Behavioral Sciences, College of Medicine, Chang Gung University, Taoyuan 33302, Taiwan; ^4^Department of Neurology and Dementia Center, Taoyuan Chang Gung Memorial Hospital, Taoyuan 33305, Taiwan; ^5^Department of Neurology, Chang Gung Memorial Hospital, Linkou, and Chang Gung University College of Medicine, Taoyuan 33305, Taiwan; ^6^Neuroscience Research Center, Chang Gung Memorial Hospital, Linkou, Taoyuan 33305, Taiwan; ^7^Healthy Aging Research Center, Chang Gung University, Taoyuan 33302, Taiwan; ^8^Department of Neurological Surgery, Tri-Service General Hospital, National Defense Medical Center, Taipei 11490, Taiwan; ^9^School of Physical Therapy and Graduate Institute of Rehabilitation Science, College of Medicine, Chang Gung University, Taoyuan 33302, Taiwan; ^10^Department of Life Science, National Taiwan University, Taipei 10617, Taiwan

## Abstract

**Background:**

Parkinson's disease (PD) is the second most prevalent neurodegenerative disorder affecting 7–10 million individuals. The pathologic hallmark of PD is nigrostriatal dopaminergic neuron loss, leading to several motor and nonmotor disturbances, such as akinesia, gait disturbance, depression, and anxiety. Recent animal studies have demonstrated that physical exercise improves behavioral and neuropathological deficits in PD. However, the exact underlying mechanism underlying this effect remains unclear. In this study, we investigated whether long-term exercise has neuroprotective effects on dopaminergic nigrostriatal neurons and whether it further alleviates impairment of the gait pattern, locomotor activity, akinesia, and anxiety-like behavior in PD rats.

**Methods:**

A hemiparkinsonian rat model, generated by unilateral injection of 6-hydroxydopamine (6-OHDA) into the medial forebrain bundle, was applied to evaluate neuroprotective effects and motor behaviors. Comprehensive spatiotemporal gait analysis, open-field locomotor activity, akinesia, apomorphine-induced rotational analysis, and dopaminergic neuron degeneration level were assessed every week and up to 8 weeks after daily voluntary running wheel exercise.

**Results:**

Compared with the sham-treated group, we found that 10 weeks of voluntary exercise (i.e., 2-week exercise before PD lesion and 8-week exercise post-PD lesion) significantly reduced 6-OHDA-induced motor deficits in the gait pattern, akinesia, and rotational behavior in the exercise group. Immunohistochemically, a tyrosine hydroxylase-positive neuron in the substantia nigra was significantly preserved in the exercise group.

**Conclusions:**

Our results demonstrated that long-term exercise training is effective for neuroprotection and further attenuates motor declines induced by 6-OHDA in an experimental model of PD. Our data further highlighted potential therapeutic effects of long-term physical exercise relevant to clinical effects for further potential application on human PD subjects.

## 1. Introduction

Parkinson's disease (PD) is an idiopathic disease of the nervous system characterized by progressive tremor, bradykinesia, rigidity, and postural instability. The pathologic hallmark of this movement disorder is due to the denervation of dopaminergic neurons in the nigrostriatal pathway, leading to both motor and nonmotor symptoms [1–3]. Pharmacological intervention, administered through dopamine supplementation (e.g., levodopa) or dopamine agonist, has beneficial effects on the improvement of motor symptoms, with long-term dopaminergic replacement therapy; however, several, mainly motor, complications such as levodopa-induced dyskinesia and motor fluctuations are common side effects after 5–10 years of levodopa administration [4–6]. The development of novel treatments could be important for reducing or slowing down the progressive neurodegeneration of dopaminergic neurons and eliminating pharmacologic complications that are a significant challenge for clinical practitioners for PD therapy.

Physical exercise is a common, practical, uncomplicated, noninvasive, and relatively safe approach for improving motor and cognitive functions and dopaminergic functioning in clinical PD patients [7, 8]. Previous literature strongly suggested that exercise is useful in forestalling the onset of PD and slowing its progression, as prompted and recommended by clinical practitioners to their patients. Furthermore, physical activity reduced the incidence of PD and improved the initiation of movement, balance, and other physical functions in PD patients [9–13]. Regarding nonmotor symptoms of PD, earlier research also indicated that motor memory, cognitive ability, and daily activity can be improved after aerobic exercise training in individuals with PD [10, 14–17]. Although these empirical data encourage exercise interventions for PD patients, epidemiological studies still cannot distinguish between beneficial impacts of exercise-induced neuroprotective effects on PD patients with and without appropriate exercise [18]. Moreover, previous prospective studies have been brief and underpowered, lacked proper controls, and failed to differentiate disease progression between short-term symptomatic improvement and long-term functional recovery in the PD population. To overcome these difficulties in human studies, diseased animal models could provide a unique platform to eliminate theoretical discrepancy and clarify the necessary adjustments of an effective therapeutic strategy in understanding beneficial effects and related mechanisms of exercise training protocols and intervention.

6-Hydroxydopamine (6-OHDA) is the most commonly used neurotoxin to induce PD in the rodent model [19, 20]. With a unilateral intracortical infusion of 6-OHDA into the substantia nigra (SN), medial forebrain bundle (MFB), or striatum (Str) of experimental animals, such animal models can induce hemiparkinsonian symptoms such as akinesia, bradykinesia, and gait disturbance, which are similar to those in humans with PD [17, 19, 21, 22]. Although studies have reported that voluntary exercise can improve motor dysfunctions in 6-OHDA models of PD [7, 19], information regarding time frame changes during disease progression and motor behaviors following a voluntary exercise intervention in PD animal study is still insufficient. Furthermore, the intensity and duration of exercise training required to exert effects on dopaminergic function remain undefined [17, 23].

The present study identified the therapeutic potential of long-term effects of early voluntary exercise intervention on a PD animal model by monitoring gait, locomotor activity, akinesia, and dopaminergic nigrostriatal neurons in 6-OHDA hemiparkinsonian rats as an early step toward possible eventual clinical conditions. Therefore, strategies to investigate individual variability in lesion and injury progression may further play a pivotal role in evaluating therapeutic effects of voluntary exercise on the 6-OHDA study model.

## 2. Materials and Methods

### 2.1. Animals

Male Sprague-Dawley rats (8 weeks; BioLASCO Taiwan Co., Ltd., Taiwan) were housed in a temperature- (22 ± 2°C) and humidity-controlled (50%) environment and placed on a 12/12 h reversed dark-light cycle with ad libitum access to standard chow and tap water. Upon arrival, the rats were allowed to have 2 weeks of the acclimation period to the animal facility. After the acclimation period, the rats were allocated randomly and equally to two groups: experimental and control groups. All procedures were approved by the Institutional Animal Care and Use Committees of Chang Gung University (approval no. CGU16-031) and performed following the Guide for the Care and Use of Laboratory Animals 8th edition (2011) for humane treatment at all times of the present study.

### 2.2. Parkinsonian Rat Model

Animals received 6-OHDA injection when the body weight increased to 350 g. Anesthesia was administered through an intraperitoneal injection of Zoletil (50 mg/kg, Vibac, France) mixed with xylazine (10 mg/kg, Rompun, Bayer, Germany). The anesthetized rats were placed in a stereotaxic frame (Stoelting, Wood Dale, IL, USA) for the injection procedures. A longitudinal incision was made along the dorsal medial surface of the head, exposing the skull from the bregma to the lambda. A 1.5 mm diameter hole was drilled using a stereotaxic high-speed drill at the following locations: −4.3 mm (AP to bregma), 1.6 mm (ML), and −8.2 mm (DV), to the MFB. Rats with MFB lesions received an injection of 8 *μ*g 6-OHDA (Sigma-Aldrich) dissolved in 4 *μ*l of 0.02% ascorbic saline into the MFB of the left hemisphere, causing a progressive retrograde and dose-dependent neuronal degeneration in the nigrostriatal pathway [19, 24]. Injection was administered at the rate of 0.5 *μ*l/min by using a 10 *μ*l Hamilton microsyringe fitted with a 26-gauge steel cannula and left for 8 min to assure the solution diffusion to prevent backflow. The wound was closed after the cannula was removed.

### 2.3. Running Wheel Apparatus and Intervention Procedures

The running wheel for rodents was applied for exercise intervention in the present study. The exercise rat was caged in the customized exercise cage (Running Rat RR-6, Bio-Cando Inc., Taiwan) which contained a transparent Plexiglas arena (45.5 cm (*L*) × 23.5 cm (*W*) × 20 cm (*H*)), one running wheel (radius = 15 cm, circumference = 70.7 cm), and one rat only. The exercise animals had unlimited access to a running wheel in their exercise cage. The running wheel apparatus (radius = 15 cm, circumference = 70.7 cm) was connected to a sensor that measured the daily rotations and converted the numbers of rotations to distance (meters). The sensor outputs were recorded and analyzed to acquire and monitor the exercise performance of rats.

**Figure 1:**
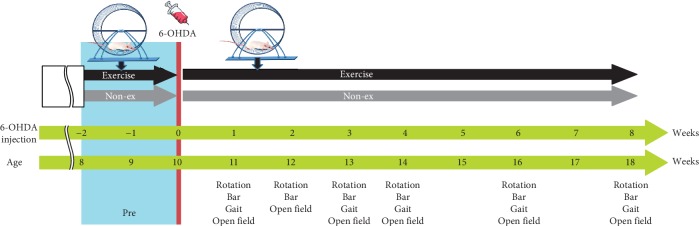
Study design for long-term effects of exercise training in 6-OHDA-induced PD rats. The performance of exercise intervention and sham control rats was assessed every day before the PD lesion and also after 8 successive weeks. Behavioral tests including the rotational behavior test, bar test for akinesia, gait test, and open-field test were performed to investigate time course treatment effects.

To examine whether voluntary running wheel exercise can alleviate functional and neurobehavioral deficits following the 6-OHDA injection, the present study designed two exercise intervention phases, before and after 6-OHDA treatment (Figure 1). In the first phase, the experimental rats, allocated to the exercise group, attended 2 weeks in their exercise cages before 6-OHDA administration. This phase was designed to establish self-sustaining exercise habits of the experimental rats. In the second phase, after 6-OHDA injection, the experimental rats were immediately placed back in their home cages for consecutive self-sustaining exercise over the next 8 successive weeks. Based on the recording of numbers of rotations in the running wheel, all experimental rats showed the voluntary physical exercise in the running wheel. Before 6-OHDA lesion, the average running distance is 3661 ± 932 m/day in phase one. After 6-OHDA lesion, the average running distance in phase two is 1292 ± 770 m/day. The control rats, allocated to the nonexercise group, were maintained under the nonexercise life pattern in their regular home cages (without a running wheel) before and after 6-OHDA administration.

### 2.4. Behavioral Testing

All behavioral tests were performed in red light conditions in a sound-attenuated and temperature- and humidity-controlled room. Four motor behavior tests, namely, gait pattern, akinesia, open field, and apomorphine-induced rotation, were performed in the same sequence during the week to test the changes in functional motor performances. The tests had at least a 24 h interval in between to avoid possible interactions. The gait analysis and open-field, rotational behavior, and akinesia tests were performed at the baseline and after intervention in weeks 1, 2, 3, 4, 6, and 8 (Figure 1).

#### 2.4.1. Gait Analysis

The gait parameters that were significantly affected by 6-OHDA lesions in the nigrostriatal pathway in rats may be used to study disease progression and to test time frame effects of voluntary exercise [7, 19]. By using the video-based gait analysis system, detailed abnormalities of spatiotemporal gait parameters accompanied by other behavioral dysfunction and immunohistochemical data can provide different methods for evaluating the therapeutic efficacy of voluntary exercise in the 6-OHDA rat model of PD. Gait analysis is a systematic assessment of quantitative changes on bodily movements during disturbed locomotion in 6-OHDA-administered rats. The procedure of gait analysis to measure locomotor functions was described previously [19]. The walking track apparatus consisted of an enclosed walkway made of transparent Plexiglas (80 cm (*L*) × 6 cm (*W*) × 12 cm (*H*)) with an inclined mirror positioned at a 45° angle beneath the walkway. With a high-speed and high-resolution camera (PX-100, JVC, Japan) placed parallelly to view the image of the entire walkway, sagittal gait characteristics, reflected by the mirror image, could be acquired for further pattern identification [19]. During the measurement, the rats were trained to walk smoothly and freely at their own pace on the walking track. After recording, image data captured from each trial was processed semiautomatically to identify the sequential footprints using MATLAB software (MathWorks, version 7.6., R2008a). Four spatial parameters, namely, step length, stride length, base of support, and foot angle, and three temporal gait parameters, namely, walking speed, stance/swing phase time, and stance/swing ratio, were determined [19, 20].

#### 2.4.2. Akinesia Test

The forelimb akinesia test, as known as the bar test, was used to evaluate the forelimb mobility in a PD rodent model [19, 25, 26]. During the bar test, each rat was placed gently on a table. Each forepaw was placed alternatively on a horizontal Plexiglas rod (0.7 cm diameter, produced in-house), which was 9 cm above the table. Total time consumption, from placing the forepaw on the bar to the first complete removal of the paw, was recorded.

#### 2.4.3. Open-Field Test

The open-field test was used to evaluate motor movement distance activity and anxiety [27–29]. It was performed in an open-topped black Plexiglas arena (60 cm (*L*) × 60 cm (*W*) × 80 cm (*H*), produced in-house), and each rat was allowed to explore the space after placing in the center of the arena for 10 min. All the activities of the animals within the arena were automatically recorded using SMART tracking software (PanLab Harvard Apparatus, Holliston, MA, USA). The behavioral patterns within the arena were analyzed based on the total movement distance: distance traveled both in the center and along the perimeter of the area, time spent in the center, movement velocity, and freezing time [30, 31]. The arena was illuminated with white light and cleaned with a 75% ethanol solution to prevent unwanted optical and odor interferences during the testing.

#### 2.4.4. Apomorphine-Induced Spontaneous Rotation

Apomorphine administration induces abnormal contralateral rotations in hemiparkinsonian model rats; these were measured every week after 6-OHDA injection to estimate the severity of dopamine depletion [19, 32, 33]. Apomorphine (0.5 mg/kg in 0.1% ascorbic acid, s.c.; Sigma) was subcutaneously administered to the 6-OHDA-lesioned rats. Five minutes after the apomorphine was administered, the rats were individually placed in a 40 cm diameter round bowl (produced in-house), and counterclockwise (contralateral) rotations were monitored using digital video camera recording for 60 min. The apomorphine-induced rotation data of each rat was automatically uploaded to a computer for further analysis.

### 2.5. Immunohistology Investigation

After voluntary exercise and behavioral tests were assessed 56 days postlesion and 70 days postvoluntary exercise, animals were sacrificed for tyrosine hydroxylase (TH) staining to evaluate and estimate dopaminergic neurons in the SN. Animals were deeply anesthetized with an overdose of inhaled isoflurane in oxygen (<0.5%) (100–300 ml/min, 1.5%–3.5%), followed by an intracardial infusion of phosphate-buffered saline (PBS) and a 4% paraformaldehyde fixative solution. Brains were carefully removed, postfixed for 3 days, and cryoprotected in 30% sucrose solution at 4°C until brains sank completely. The cerebral tissues were sectioned at a thickness of 30 *μ*m coronal blocks. The sections were selected from the Str and SN on a cryostat (Leica CM3050 S Cryostat, FL, USA). The free-floating sections were quenched with 0.3% H_2_O_2_/PBS for 10 min and 10% milk (Anchor Shape-Up, New Zealand) for 60 min to block a nonspecific antibody. Sections were then incubated with rabbit primary anti-TH (1 : 1000, AB152, Millipore, USA) for 1 h at room temperature. Subsequently, sections were washed in PBS and incubated for 1 h with a secondary anti-rabbit antibody (1 : 200, MP-7401, Vector Labs, USA) containing 3% normal horse serum in PBS. After rinsing, sections were immunostained with 3,3′-diaminobenzidine (SK-4105, Vector Labs, USA) and observed for 3–5 min. Lastly, the stained sections were mounted onto slides, air-dried, dehydrated using xylol (Sinopharm, China), cleared in xylene, and cover slipped in *DPX* mounting medium. TH-immunopositive cells in the SN pars compacta were counted manually in each section [7, 27] using a high-magnification (200x) microscope. The TH neuron count in the SN and the density of striatal dopaminergic nerve terminals were determined using light microscope Spectrum Software (Aperio ScanScope CS, Vista, CA, USA).

### 2.6. Statistical Analysis

For the statistical analysis of gait measurement, two-way repeated measures analysis of variance (ANOVA) was used to investigate parameter changes between both the groups (exercise vs. nonexercise) and time (week) factors. When the ANOVA showed significant effects, multiple within-subject comparisons with the Bonferroni post hoc analysis were performed to compare the effects of exercise and time courses. The independent *t*-test was conducted to verify the intergroup difference, and one-way ANOVA was used to investigate intragroup differences in the time course when no significant main effect after two-way repeated measures ANOVA was observed. Data were analyzed using SPSS version 24.0 (IBM Corp, Armonk, NY, USA), with the significance level set at *p* < 0.05 for each analysis. All data were presented as mean ± standard error of the mean.

## 3. Results

### 3.1. Apomorphine-Induced Spontaneous Rotation and Akinesia

**Figure 2:**
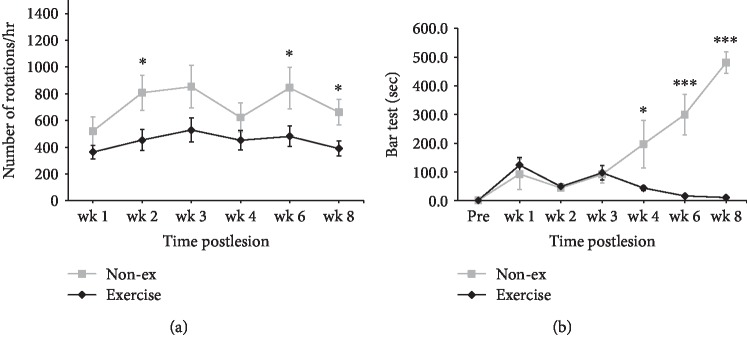
Effects of exercise on apomorphine-induced rotational behavior (a) and akinesia determined using the bar test (b) in the sham and exercise groups. Error bars = SEM, ^∗^*p* < 0.05, ^∗∗∗^*p* < 0.001, significant difference between the two groups.

In the apomorphine-induced spontaneous rotation, repeated measures data demonstrated significant effects on the group (*F* = 42.033, *p* < 0.001) and time course (*F* = 3.641, *p* = 0.005) but no significant effects on the group vs. time course interaction (*F* = 1.215, *p* = 0.309). The intergroup data for the exercise-treated group showed a significantly lower rotation number at week 2 (*p* = 0.047), week 3 (*p* = 0.043), week 4 (*p* = 0.03), week 6 (*p* = 0.014), and week 8 (*p* = 0.001) than that for the nonexercise control group (Figure 2(a)).

The bar test is a useful indicator of akinesia, and two-way repeated measures ANOVA demonstrated significant main effects on the group (*F* = 64.143, *p* < 0.001), time course (*F* = 14.756, *p* < 0.001), and group vs. time interaction (*F* = 21.821, *p* < 0.001). The intergroup analysis indicated a significantly higher bar test score at week 4 (*p* < 0.001), week 6 (*p* < 0.001), and week 8 (*p* < 0.001) in the nonexercise control group than in the exercise-treated group (Figure 2(b)).

### 3.2. Gait Performance

**Figure 3:**
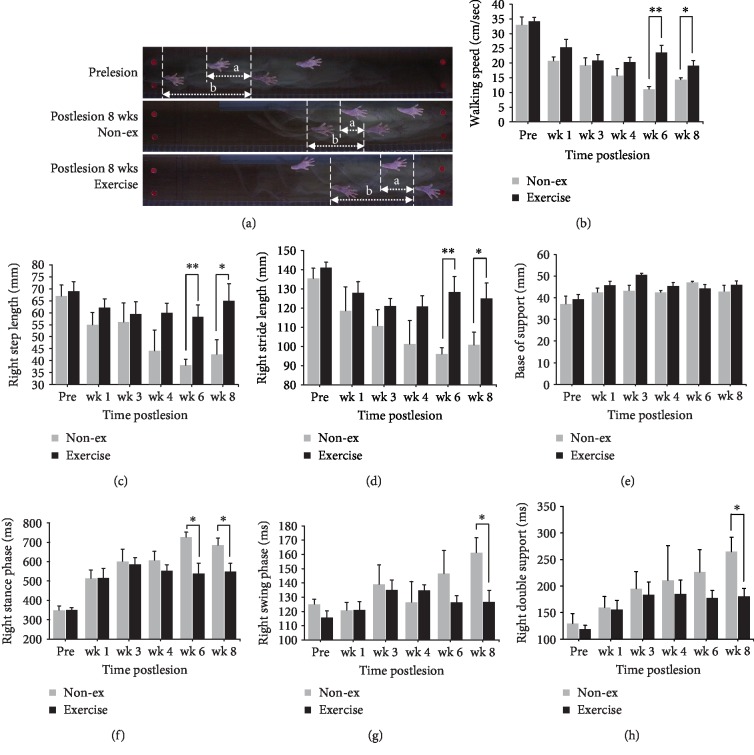
Characteristics of stepping footprint during 1 s walkway locomotion in a prelesion, an 8-week post-PD-lesioned rat with sham treatment, and an 8-week post-PD-lesioned rat following long-term exercise training (a). Time course changes of walking speed (b), step length (c), stride length (d), base of support (e), stance/swing phase time (f, g), and double support time (h) in the affected hind limb in the sham and exercise training PD rats over an 8-week observation period. Note that the walking speed, step length, and stride length decrease significantly in the sham treatment group but show a less decrease in the exercise treatment group. ^∗^*p* < 0.05, ^∗∗^*p* < 0.01, and ^∗∗∗^*p* < 0.001, significant difference between the two groups at each time point.

No significant intergroup difference in all gait parameters for the pretreated 2-week exercise group and the nonexercise control group before 6-OHDA administration was observed (*p* > 0.05). This indicated no significant difference between the exercise group and the nonexercise control group at the beginning of the present study.  Figure 3(a) shows typical footprint images recorded from an 8-week PD rat with no exercise and an 8-week PD rat with exercise. Two-way repeated measures ANOVA showed significant main effects of group on walking speed (*F* = 15.944, *p* = 0.003), step length (*F* = 6.578, *p* = 0.028), stride length (*F* = 13.176, *p* = 0.005), and stance phase time (*F* = 6.000, *p* = 0.034), suggesting less impairment of the gait pattern in the exercise group as compared with the nonexercise group. Following post hoc *t*-tests between groups in each time point revealed that this difference was largely driven by exercise effects observed starting at six weeks or seven weeks of exercise on walking speed, step length, stride length, stance phase time, swing phase time, and double support time (unpaired *t*-tests, *p* < 0.05) (Figures 3(b)–3(h)).

### 3.3. Open-Field Locomotor Activity

**Figure 4:**
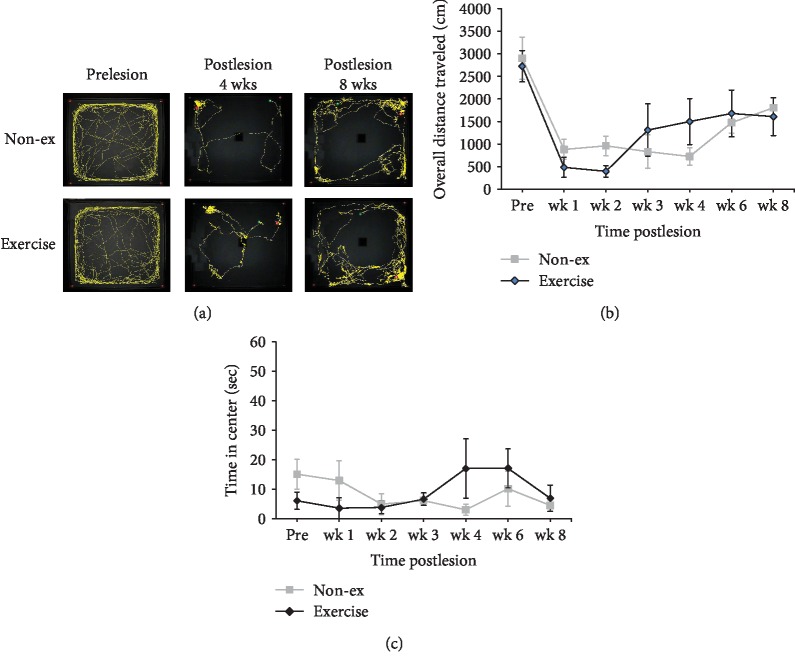
Effects of long-term exercise on locomotor activity and anxiety-like behavior, evaluated using the open-field test. (a) Representative traces of rat movement during the open-field test in the sham and exercise-treated rats at prelesion, 4 weeks postlesion, and 8 weeks postlesion. No significant differences were observed between the exercise and nonexercise groups in overall traveled distance (b) and time in the center (c) during the observation time points.

The present study also calculated the overall distance traveled using an open-field test to investigate the general locomotor activity between the 6-OHDA exercise-treated and nonexercise-treated rats (Figures 4(a) and 4(b)). In the overall traveled distance, the repeated measures data analysis demonstrated significant effects on the time (*F* = 5.363, *p* < 0.001) and not on the group (*F* = 0.006, *p* = 0.941) and group vs. time interaction (*F* = 0.566, *p* = 0.755) (Figure 4(c)). In the anxiety-like behavior measured from the total retention times in the central zone of the open field, the repeated measures ANOVA indicated no significant main effects of the time (*F* = 0.681, *p* = 0.666), group (*F* = 0.026, *p* = 0.874), and group vs. time interaction (*F* = 1.144, *p* = 0.348). There are no significant differences in overall traveled distance and times in the central zone between the exercise and nonexercise groups at each time point (all *p* > 0.05).

### 3.4. Immunohistochemistry

**Figure 5:**
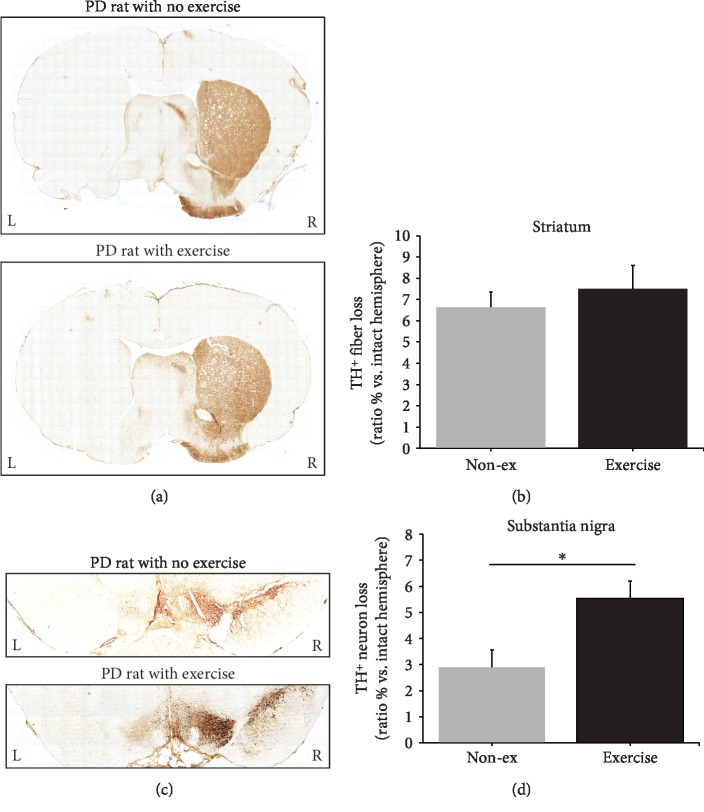
Representative tyrosine hydroxylase (TH) immunostaining in the striatum (a) and substantia nigra (c) in one nonexercise rat and one exercise rat 8 weeks post-PD lesion. No significant difference was observed in TH immunoreactivity in the striatum (b). However, a significant difference in TH immunoreactivity in the substantia nigra was observed between the nonexercise and exercise groups 8 weeks post-PD lesion. ^∗^*p* < 0.05.

TH-positive immunoreactivity in the SN and Str of the rats with 6-OHDA lesion is demonstrated in Figure 5 in the present study. Immunohistochemistry data demonstrated that MFB 6-OHDA infusion induced TH cell and fiber loss on the ipsilateral (left, L) side of the SN and the Str (Figures 5(a) and 5(c)). TH-positive immunoreactivity in the lesioned Str did not significantly increase 8 weeks after exercise intervention in the exercise-treated group (Figure 5(b)). A significant preservation in TH-positive cells in the SN was observed in the exercise-treated group compared with the nonexercise group (*p* = 0.031) (Figure 5(d)).

## 4. Discussion

The present study investigated motor behaviors in unilateral 6-OHDA-lesioned rats in response to voluntary physical exercise to better understand the interrelation of time course exercise intervention, neuroprotective effect, and motor behaviors. We observed that long-term voluntary physical exercise training restored several PD-related motor dysfunctions and alleviated the dopaminergic neuron loss in the SN in the 6-OHDA-induced PD rat model, suggesting that exercise maintains motor function and potentially reduces dopaminergic neuron damage.

Based on our detailed assessment by using the bar and rotation tests, the decrease in dopaminergic function caused by unilateral 6-OHDA MFB lesions can be investigated quantitatively and objectively [28, 29]. The rotational behavior data of the nonexercise control group increased at week 2, then sustained in the plateau continuing through week 6, and further extended to week 8 in the present study, but such increased rotation was not found in the exercise-treated group. Similarly, the bar test for akinesia showed a significantly higher parkinsonian value at week 4 and a gradual increase till week 8 in the nonexercise control group but not in the exercise-treated group. Moreover, the immunohistochemistry analysis showed more preserved dopaminergic neurons in the exercise-treated group than in nonexercise control group, suggesting a neuroprotective effect of exercise intervention. All these findings suggest that voluntary exercise was sufficient to reduce neurotoxic effects of 6-OHDA and protect DA neurons, which confirms the preliminary findings of the present study that voluntary exercise is neuroprotective and agrees with the findings of the published data [7, 34]. This neuroprotective effect correlated well with the recovery of functional activity (e.g., gait pattern measured by spatiotemporal gait parameters).

The present spatiotemporal gait analysis confirmed previous evidence suggesting that the PD rats with voluntary exercise in the exercise-treated group displayed a significantly higher walking speed, associated with longer step and stride lengths, than those in the nonexercise group. The walking speed of the exercise-treated group increased gradually and reached a stable plateau accompanying time course exercise intervention, which indicated that voluntary exercise improves motor function in PD animals. Moreover, these behavioral findings in the group treated with 8 weeks of voluntary exercise, accompanied by attenuation of 6-OHDA-induced degeneration of nigral dopaminergic neurons and the preservation of TH-positive fibers of the nigrostriatal pathway, strongly suggest exercise-induced neuroprotection, which has been reported in other studies [17, 35, 36]. The exercise-induced neuroprotection effect correlates with the performance in walking speed and other gait parameters after voluntary exercise intervention. By contrast, the walking speed of nonexercise 6-OHDA-injured animals significantly reduced in association with the time course change, and impairments in regulating and adjusting appropriate step and stride lengths in the affected hind limb of hemiparkinsonian rats might be attributed to dopaminergic neuron lesions in the SN and further influence the walking performance [19, 32]. In the present study, a significantly lower walking speed was observed at week 8 in both the groups, especially in the 6-OHDA exercise-treated rats, than the speed before 6-OHDA lesion. The data of the 6-OHDA nonexercise group revealed a progressively and sustained decrease in walking speed, swing phase, stance phase, and step and stride lengths compared with those of the 6-OHDA exercise-treated rats during the entire experimental period. Although the SN of the intact hemisphere did not experience dopamine depletion, the gait performance level of the unaffected hind limb also showed mild impairment, which has been reported in previous studies using kinematic analysis [19, 20, 32]. The decrease in the walking speed in 6-OHDA exercise-treated rats might also indicate that their intact hind limbs play an essential role in the gait compensatory mechanism during locomotion, carrying more weight to support the unaffected side during propulsion [37].

The present results suggest that time course voluntary exercise can have long-lasting effects on neuroprotection of dopaminergic neurons in the SN. These results are consistent with those of earlier PD animal studies, showing that physical exercise can reduce behavioral and neuropathological effects directed toward the DA neurons of the SN [38, 39]. This may involve several mechanisms such as upregulation of neurotrophic factors and a decrease in reactive oxygen species [17], mitochondrial energy metabolism [18], increased antioxidative stress activity [40], anti-inflammatory action [36, 41], enhanced synaptogenesis [18], increased neuroplasticity [17], and increased DA receptors in the SN [7, 42]. Although precise mechanisms of the neuroprotective effects of exercise on PD are still unclear, our results suggested that daily exercise intervention may not only improve motor functions but also have neuroprotective effects on dopaminergic neurons and reduce neurotoxin-induced damage on the dopaminergic neurons.

In the present study, rats were exercising when the behavioral tests were performed, and upregulation of DA receptors through exercise may have reduced the interhemispheric asymmetry following 6-OHDA lesion, thereby reducing apomorphine-induced rotations and increasing the walking speed in exercise-treated rats. These findings suggested that the long-lasting effects of voluntary exercise intervention may lower PD influences in the present study in the PD rodent model. Furthermore, this requires careful interpretation of findings from animal studies regarding any potential role of neurogenesis in the effects of exercise on PD patients. One of the main future challenges will be to assess and define exercise-induced effects on animal models comprehensively and to translate these results to human studies meaningfully.

## 5. Conclusions

In summary, the results of the present study revealed the effects of 8 weeks of voluntary exercise on motor behavior and neuroprotection in the 6-OHDA rodent model. The neuroprotective effects of voluntary exercise in the present unilateral 6-OHDA rat model accompanied the motor performance in decreasing the akinesia behavior and increasing the gait performance. In association with the immunohistological results, the decrease in dopaminergic system deterioration, as observed by TH levels, indicated protective effects of voluntary exercise on dopaminergic cells after 6-OHDA injury. The present results suggest that voluntary exercise may provide long-term improvements in functional motor behavior in PD patients.

## Data Availability

The data generated and analyzed during the current study are available from the corresponding author on reasonable request.
